# Attachment promoting compounds significantly enhance cell proliferation and purity of bovine satellite cells grown on microcarriers in the absence of serum

**DOI:** 10.3389/fbioe.2024.1443914

**Published:** 2024-11-01

**Authors:** Vincent Bodiou, Anitha Ajith Kumar, Edoardo Massarelli, Tessa van Haaften, Mark J. Post, Panagiota Moutsatsou

**Affiliations:** ^1^ Mosa Meat BV, Maastricht, Netherlands; ^2^ CARIM (The Cardiovascular Research Institute Maastricht), Faculty of Health, Medicine and Life Sciences, School for Cardiovascular Diseases, Maastricht University, Maastricht, Netherlands

**Keywords:** cultivated meat, bovine myoblasts, Microbeads, cell expansion, proliferation, bioprocessing, cell attachment, coating

## Abstract

**Introduction:**

To bring cultivated beef to the market, a scalable system that can support growth of bovine satellite cells (bSCs) in a serum-free and preferably also animal-free medium is of utmost importance. The use of microcarriers (MCs) is, at the moment, one of the most promising technologies for scaling up. MCs offer a large surface to volume ratio, they can be used in scalable stirred tank bioreactors, where the culture conditions can be tightly controlled to meet the cells’ requirements (temperature, pH, dissolved oxygen). The inherent capacity of the cells to migrate from one MC to another, also known as bead-to-bead transfer, facilitates a scale-up strategy involving MCs. Previous studies have shown growth of bSCs on three commercially available MCs in serum containing media. Unfortunately there is currently no information available regarding their growth on MCs in serum-free conditions.

**Methods:**

In this study, we aimed to find suitable serum-free media, MCs and attachment promoting compounds (APCs) supporting the growth of bSCs. Initially, six commercial MCs and three serum-free media were evaluated. The effects of three APCs were compared (vitronectin, laminin and fibronectin). Subsequently, the effects of different concentrations and modes of addition of the best performing APC were investigated.

**Results and Discussion:**

Our results showed that Cytodex 1, Synthemax II and CellBIND supported bSCs’ growth in all serum-free media. Overall, better growth was observed with Cytodex 1 in serum-free proliferation media. We showed that the use of laminin or vitronectin with Cytodex 1 can significantly improve cell growth and purity. Laminin also allowed attachment and growth of bSCs on Plastic MCs which had been previously unsuccessful without APCs. Finally, we optimized the use of vitronectin from a sustainability and process perspective, and showed that it can be used solely as a coating for Cytodex 1 (16–100 ng/cm^2^) MCs, instead of as a medium supplement, enhancing cell attachment and proliferation.

## 1 Introduction

Bioprocess scale-up plays a key role towards the commercialization of cultivated meat (Moutsatsou et al., 2023; Post et al., 2020). A successful bioprocess should consistently and predictably generate a product, while using as few resources as possible (personnel, space, time, equipment, energy, consumables). Scale-up of bacterial, fungi, yeast and in particular mammalian cell cultures have been extensively described and can be used as reference for cultivated meat (Allan et al., 2019; O’Brien and Hu, 2020; Singhania et al., 2022; Tripathi and Shrivastava, 2019). However, contrary to the typical bioprocesses for the production of biologics, where cells usually grow as single cell suspension and the product of interest is synthesized by the cell, in the context of cultivated meat, the cultivated cells are adherent and constitute the final product, rendering the scale-up strategy more challenging (Cameron et al., 2023; Lee et al., 2022). Satellite cells (SCs) are found in skeletal muscles of various animals (mammals, birds, fish, crustaceans, etc.) and are myogenic precursors involved in muscle tissue regeneration (Hartley et al., 1992; Koumans and Akster, 1995; Mauro, 1961). Because of their muscle lineage commitment and ability to proliferate, SCs are often used for muscle tissue production (Reiss et al., 2021). Stout et al. (2023) were able to achieve up to 45 population doublings with primary bSCs, while in our group, 20 to 40 population doublings are typically reached (data not shown). Although a decrease in growth rate is usually observed after ∼25 population doublings, considering 3 millions cells as an initial number (usual number of cells isolated from a donor), a 315 L or 3.30 × 10^5^ m^3^ production capacity can be achieved with 20 or 40 population doublings, respectively, calculating with a 10 million cells/mL final cell density. Under the appropriate conditions, they have the ability to either self-renew or differentiate into muscle cells, meeting both the upstream and downstream requirements (biomass and tissue generation respectively). The use of microcarriers (MCs) has been recommended as an established method for upscaling the expansion of bSCs (Bodiou et al., 2020). MCs can be used as anchoring points for the cells and due to their low specific gravity can be suspended in a bioreactor, offering a large surface/volume ratio. They are also versatile as they can be used in combination with a lot of different bioreactor types, such as stirred-tanks, packed-beds, fluidised-beds and air-lift bioreactors.

In previous studies (Andreassen et al., 2022; Verbruggen et al., 2018), bSCs were successfully cultured on three commercially available animal material-free MCs, CellBIND, Cytodex 1 and Synthemax II. However, all these studies were performed in serum-containing media. Serum contains a multitude of compounds, such as growth factors, cytokines, vitamins and proteins, and is widely used in cell culture as its rich composition can promote cell attachment, proliferation and/or differentiation (Lee et al., 2022; Subbiahanadar Chelladurai et al., 2021; Zheng et al., 2006). Because of its ethical and scientific controversies though (Kolkmann et al., 2022; Stout et al., 2022), using it in the context of cultivated meat is avoided. It is therefore necessary to find MCs that can support growth of bSCs in a serum-free medium. Many other types of primary stem cells have been successfully cultured on MCs in both serum and serum-free media (Boudreault et al., 2001; Chen et al., 2011; Molnar et al., 1997; Norris et al., 2022; Rozwadowska et al., 2016; Shahar et al., 1992; Van Beylen et al., 2021; Zernov et al., 2022). Despite some differences, bSCs share features with them, such as attachment dependency, proliferation rate, stemness and the results might therefore be translatable. It is for instance well known that for adherent stem cells, extracellular matrix (ECM) proteins can be used in combination with serum-free media to enhance cell attachment (Stout et al., 2022).

Cell attachment is a crucial step for the proliferation and differentiation of many anchorage-dependent cell types, including SCs (Berrier and Yamada, 2007; Huang and Ingber, 1999; Yin et al., 2013). Cell adhesion molecules are groups of proteins (integrins, cadherins, selectins, members of the immunoglobulin superfamily) which are located on the cells’ surface and play important roles in cell-cell as well as cell-matrix interactions (Alberts et al., 2002; Elangbam et al., 1997). Integrins are the main proteins involved in cell-matrix attachment. They are transmembrane receptors consisting of two subunits (α and β) which, depending on their variant, present different affinity for proteins (Takada et al., 2007). For instance α7β1 integrins bind specifically to laminin, α5β1 is specific to fibronectin, whereas αvβ3 is specific to vitronectin (Barczyk et al., 2010; Rozo et al., 2016). A previous publication showed that bSCs express α3, β1 and α5 integrins (Wang et al., 2020) and β1 integrin has also been found to promote proliferation and differentiation of bSCs (Pang et al., 2019; Wang et al., 2020). In mouse SCs, a high expression of β3 integrin was observed during proliferation (Liu et al., 2011). For bSCs therefore, the use of laminin, fibronectin or vitronectin could be beneficial. Moreover, and despite their difference in affinity, it has been shown that various integrins (8 out of the 24 families identified so far) can bind to the sequence arginine-glycine-aspartic acid, also called RGD sequence (Kapp et al., 2017; Takada et al., 2007). This sequence is found in many ECM proteins (collagen, laminin, vitronectin or fibronectin), which are therefore widely used *in vitro* cultures to enhance cell adhesion (DeQuach et al., 2010). In contrast to serum-containing cultures where an abundance of proteins and attachment compounds are present in the serum, especially vitronectin, serum-free cultures typically require the use of attachment promoting compounds (APCs) and other molecules to compensate for the absence of serum (Dessels et al., 2016; Subbiahanadar Chelladurai et al., 2021). Animal proteins are commonly used as APCs, however with the rise of cellular agriculture and the need for more ethical, reproducible and cost-efficient bioprocesses, research on plant and algae derived proteins and peptides has emerged, however it is still at nascent stage (Hubalek et al., 2022; Kong et al., 2023; Kong and Huang, 2023; Melzener et al., 2023; Seo et al., 2023; Teo et al., 2023). In the last years many efforts have gone towards developing chemically-defined serum-free media for bSCs (Kolkmann et al., 2020; Kolkmann et al., 2022; Messmer et al., 2022; Stout et al., 2022). Nonetheless, research has been mainly focused on static 2D environments and the influence of APCs in serum-free media still has to be evaluated in dynamic MC based cultures.

In this study, we aimed to find suitable serum-free media, MCs and APCs supporting the attachment and growth of bSCs on MCs in agitated suspension cultures. In the first step, we screened several MCs and media in the absence of APCs in order to find the most promising candidates. Then, we focused on the use of APCs to enhance attachment and subsequent cell growth. Finally, the effects of APC concentration and mode of addition on proliferation and differentiation were investigated.

## 2 Materials and methods

### 2.1 Cell isolation and purification

bSCs were isolated as previously described (Ding et al., 2018; Messmer et al., 2022). Briefly, skeletal muscle obtained from slaughtered cattle (male and female, aged from 1 to 7 years) was chopped prior digestion with collagenase (1 h at 37°C) (CLSAFA, Worthington). Larger pieces of tissues were filtered out using 100 µm nylon mesh cell strainer. Red blood cells contained in the filtrate were lysed with ammonium–chloride–potassium lysis buffer (A9434, Sigma-Aldrich; 237205, Sigma-Aldrich) (1 min at 24°C) and remaining cells were filtered using 40 μm nylon mesh cell strainer. The obtained suspension was then cultured in serum or serum free proliferation medium for 72 h at 37°C, 5% CO_2_. Finally, purification of bSCs was performed using fluorescence-activated cell sorting on a FACSAria Fusion Cell Sorter (BD Biosciences). For that, cells were stained using NCAM1-PE-Cy7 (335826, BD Biosciences), CD29-APC (B247653, BioLegend), CD31-FITC (MCA1097F, Bio-Rad) and CD45-FITC (MCA2220F, Bio-Rad) and sorted by gating for the CD31/CD45–, CD29+/NCAM1+ population.

### 2.2 Media

#### 2.2.1 Serum-free proliferation media

**FIGURE 1:**
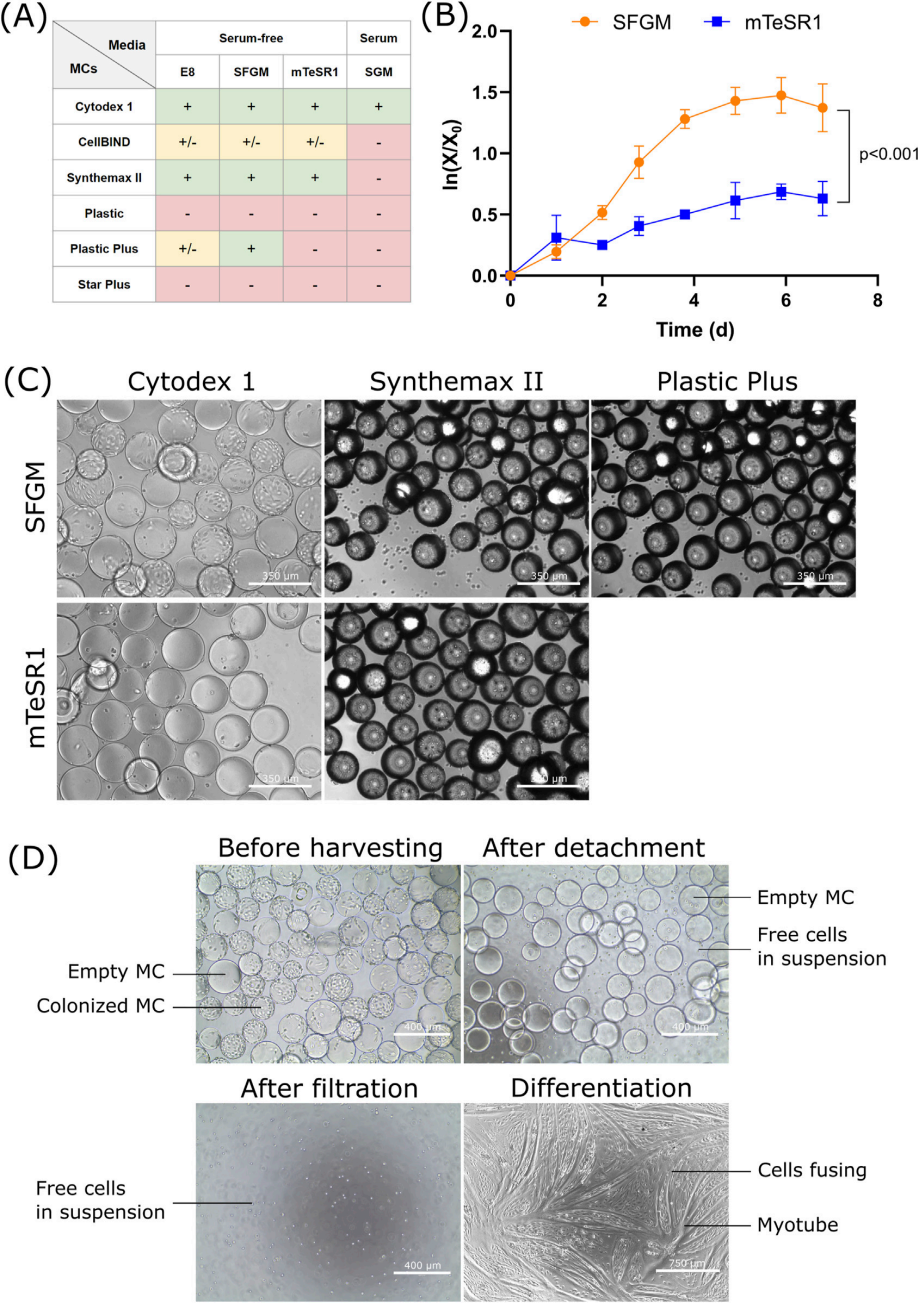
Screening of media and MCs for the growth of bSCs **(A)** Semi-quantitative results obtained from day 5 fluorescent images (Hoescht/EthD-1; Supplementary Figure S1) of bSCs grown in one serum-containing (SGM) and three serum-free media (E8, SFGM and mTeSR1) and on six different MCs (Cytodex 1, CellBIND, Synthemax II, Plastic, Plastic Plus and Star Plus). Symbols: (−): cells failed to attach; (+/−): cells attached but showed poor spreading or growth; (+): cells attached, spread and grew. **(B)** Logarithmic growth curve (n = 3) and **(C)** Day 6 bright field images (magnification ×10; scale bar = 350 µm) of bSCs grown in two serum-free media (SFGM and mTeSR1) and on three different MCs (Cytodex 1, Synthemax II and Plastic Plus). The experiment was performed in 30 mL spinner flasks at 10 cm^2^/mL and seeding of 5,000 cells/cm^2^. Significant difference between SFGM and mTeSR1 was found by analysis of covariance (*p* < 0.001). **(D)** Bright field images (magnification ×10, scale bar = 400 μm; or magnification ×4, scale bar = 750 µm) of the harvesting process of bSCs grown on Cytodex 1 in SFGM. The harvesting was performed in a 30 mL spinner flask.

**FIGURE 2:**
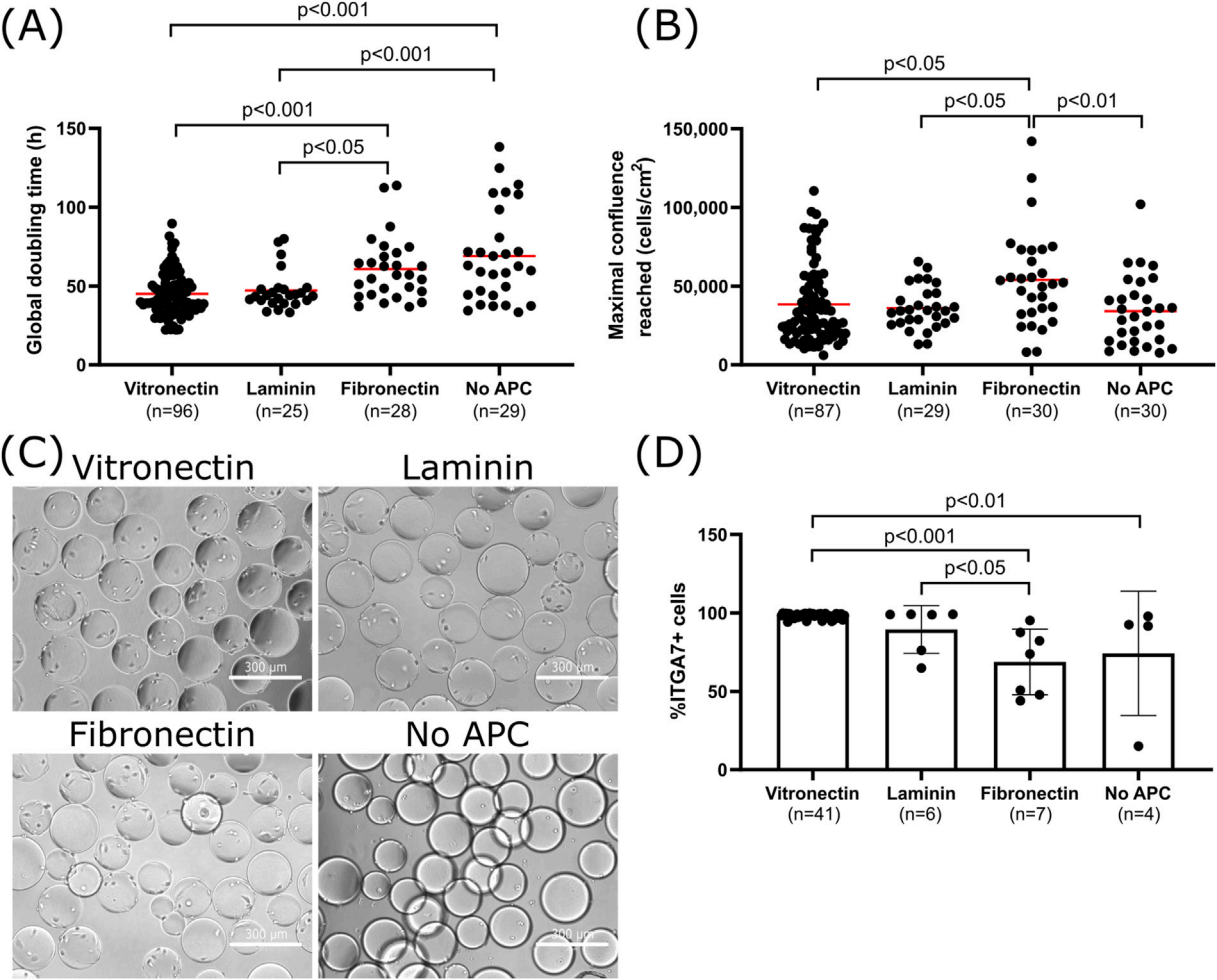
Effect of APCs on cell attachment, growth and purity of bSCs **(A)** Global doubling time at maximal cell density. **(B)** Maximal confluence reached for bSCs grown on Cytodex-1 MCs coated with vitronectin, laminin, fibronectin or without APC in SFGM in 30–100 mL spinner flasks (n = 189 observations in total from 27 different donors; Vitronectin: n = 100 from 15 donors; Laminin: n = 29 from 5 donors; Fibronectin: n = 30 from 8 donors; No APC: n = 30 from 16 donors; Red bars represent the average). Tukey’s multiple comparisons tests showed significant differences in the global doubling time between vitronectin/laminin and no APCs (Vitronectin/No APC: *p* < 0.001; Laminin/No APC: *p* < 0.001) as well as between vitronectin/laminin and fibronectin (Vitronectin/Fibronectin: *p* < 0.001; Laminin/Fibronectin: *p* < 0.05). Significant differences in maximal confluence reached were also observed between fibronectin and the other conditions (Fibronectin/Vitronectin: *p* < 0.05; Fibronectin/Laminin: *p* < 0.05; Fibronectin/No APC: *p* < 0.01). **(C)** Day 1 bright field images (magnification ×10; scale bar = 300 µm) of bSCs grown on Cytodex 1 MCs coated with vitronectin, laminin, fibronectin or without APC in SFGM in 30 mL spinner flasks. **(D)** Comparison of the bSCs purity after being cultured on Cytodex 1 MCs coated with vitronectin, laminin, fibronectin or without APC, in SFGM in 30–100 mL spinner flasks (In total n = 58 observations; Vitronectin: n = 41; Laminin n = 6; Fibronectin n = 7; No APC n = 4; biological replicates). Tukey’s multiple comparisons tests showed significantly higher purity when vitronectin is used compared to Fibronectin/No APC (Vitronectin/Fibronectin: *p* < 0.001; Vitronectin/no APC: *p* < 0.01), and also laminin compared to fibronectin (Laminin/Fibronectin: *p* < 0.05).

**FIGURE 3:**
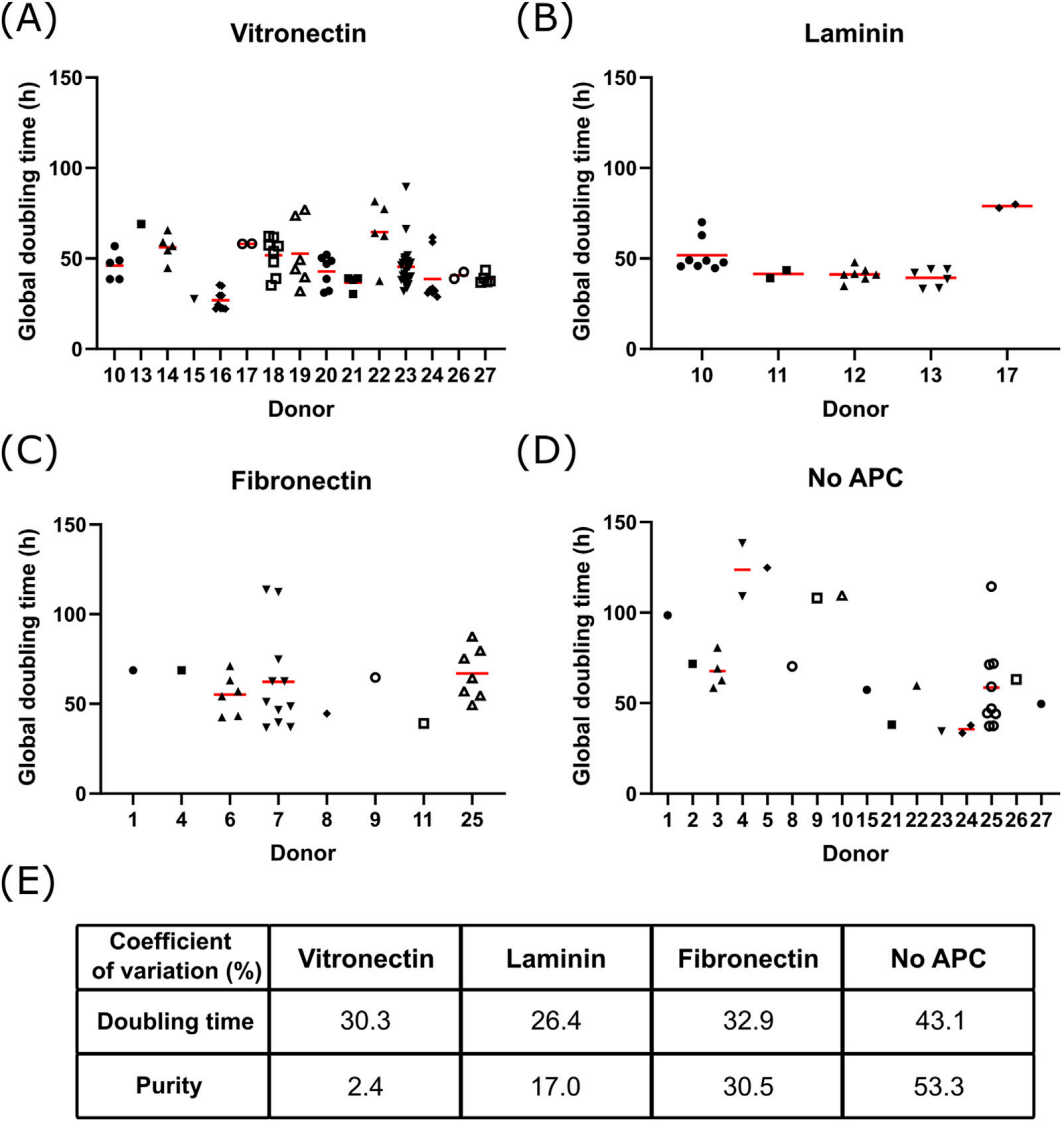
Donor to donor variability Global doubling time at maximal cell density for different donors of bSCs grown on Cytodex-1 MCs coated with **(A)** vitronectin (n = 100 from 15 donors), **(B)** laminin (n = 29 from 5 donors), **(C)** fibronectin (n = 30 from 8 donors) or **(D)** without APC (n = 30 from 16 donors); Red bars represent the average. Analysis of the variance on the donor and APC variables showed significant contribution of the APC (*p* = 0.042) and the donors (*p* < 0.0001) on the global doubling time. **(E)** Coefficient of variation of the global doubling time and purity of bSCs grown on Cytodex-1 MCs without APC or coated with vitronectin, laminin or fibronectin.

**FIGURE 4:**
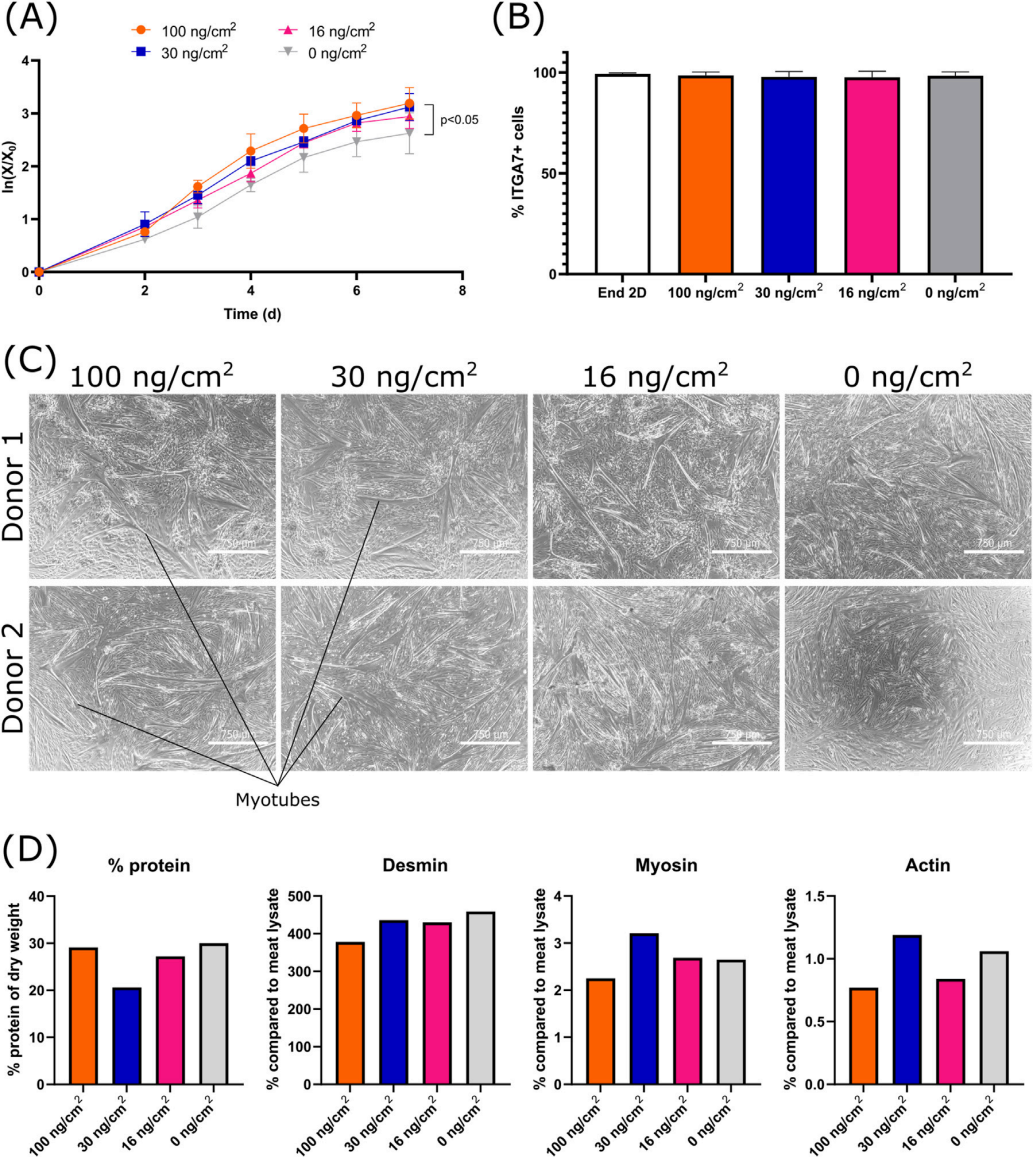
Effect of the vitronectin concentration (0, 16, 30 and 100 ng/cm^2^) during MC coating, on the growth, purity and differentiation of bSCs **(A)** Logarithmic growth. Significant difference between 0 μg/cm^2^ and 100 μg/cm^2^ was found by analysis of covariance (0 ng/cm^2^/100 ng/cm^2^: *p* < 0.05) (biological replicates n = 3). **(B)** Comparison of the percentage of ITGA7+ cells before and after cell growth for each condition (biological replicates n = 3). **(C)** Bright field images (magnification ×4; scale bar = 750 µm) of the 2D differentiation (matrigel coated) of two donors. **(D)** Comparison of the protein % of dry weight, the percentage of desmin, myosin and alpha-actin-1 (compared to meat lysate) after 3D differentiation (n = 1).

**FIGURE 5:**
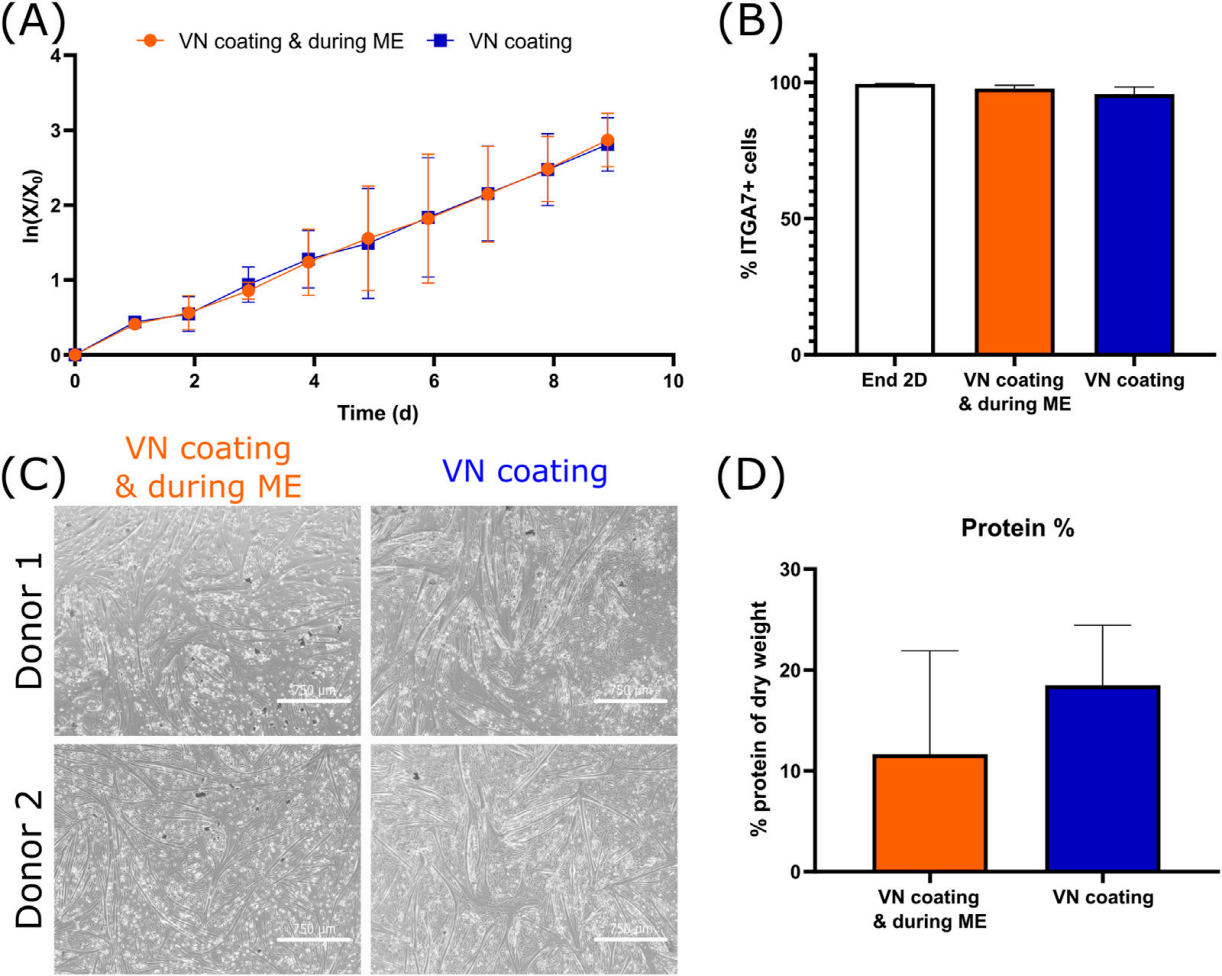
Effect of the removal of vitronectin (VN) during medium exchange (ME) on the growth, purity and differentiation of bSCs **(A)** Logarithmic growth (n = 3 biological replicates). **(B)** Comparison of the percentage of ITGA7+ cells after end of 2D seed train and after suspension growth (n = 3 biological replicates). **(C)** Bright field images (magnification ×4; scale bar = 750 µm) of the 2D differentiation of two donors. **(D)** Comparison of the protein % of dry weight after 3D differentiation (n = 2 biological replicates).

Three serum-free proliferation media were used. Two are commercially available: Essential 8™ Medium (A1517001, Gibco™, Thermo Fisher Scientific) and mTeSR1™ (85,850, Stemcell Technologies). The third one was developed specifically for bSCs by Kolkmann et al. (2022). It is composed of DMEM/F-12 (P04-041262B, PAN Biotech), 1 μg/mL α-linolenic acid (L2376, Sigma-Aldrich), 10 ng/mL bFGF-2 (100-18B, Peprotech), 50 ng/mL bHGF (100-39H, Peprotech), 5 mg/mL BSA (A9418, Sigma-Aldrich), 17.7 mM D-glucose (G7021, Sigma-Aldrich), 2 mM Glutamax (35050061, Thermo Fisher Scientific), 36 ng/mL hydrocortisone (H0888, Sigma-Aldrich), 100 ng/mL IGF-1 (100-11, Peprotech), 1% ITSE (00-101, Biogems), 155 µM Vitamin C (A8960, Sigma-Aldrich), 5 ng/mL LIF (300-05, Peprotech), 10 ng/mL PDGF-BB (100-14B, Peprotech), 10 ng/mL VEGF (100-20, Peprotech). In this article, we will refer to Essential 8™, mTeSR1™ and the in-house one as E8, mTeSR1 and serum-free growth medium (SFGM), respectively. All serum-free proliferation media were supplemented with 1% Penicillin-Streptomycin-Amphotericin B (17-745E, Lonza). For the screening of media and MCs (Figure 1), SFGM was used without fibronectin or any other APC. In the follow-up experiments (Figures 2–5) vitronectin, laminin or fibronectin was used as an additive to the SFGM formulation.

#### 2.2.2 Serum-containing proliferation medium

For serum-containing cultures, Ham’s F-10 Nutrient Mix (11550043, Gibco™, Thermo Fisher Scientific) supplemented with 20% heat inactivated fetal bovine serum (10500064, Gibco™, Thermo Fisher Scientific), 5 mM L-glutamine (17-605E, Lonza), 5 ng/mL bFGF (233-FB-025, R&D Systems) and 1% Penicillin-Streptomycin-Amphotericin B (17-745E, Lonza) was used. In this article, we will refer to this medium as serum growth medium (SGM).

#### 2.2.3 Serum-free differentiation medium

Serum-free differentiation medium (SFDM) developed by Messmer et al. (2022) was used to differentiate cells in 2D and 3D environments. It is composed of DMEM (A14430-01, Gibco™, Thermo Fisher Scientific), 5.55 mM glucose (G7021, Sigma-Aldrich), 2 mM GlutaMAX™ (35050061, Thermo Fisher Scientific), 0.5 mM sodium pyruvate (P2256, Sigma-Aldrich), 10 ng/mL EGF1 (IK0100, ORF Genetics), 0.5 mg/mL human serum albumin (RHAC-NW, Richcore), 40 μM L-ascorbic acid 2-phosphate (A8960, Merck), MEM amino acids solution (11130-051, Thermo Fisher Scientific), 6.5 mM sodium bicarbonate (S5761, Merck), 1% Penicillin-Streptomycin-Amphotericin B (17-745E, Lonza), 80 nM sodium selenite (S5261, Sigma-Aldrich), 1.8 µM insulin (P-2701000, PAN Biotech), 1 µM lysophosphatidic acid (72,694, Stemcell Technologies), 135 nM transferrin (10-366, Biogems) and in addition for 3D differentiation, 10 µM acetylcholine (A2661, Merck).

### 2.3 Planar cultures

Cells were seeded at 1,800–5,000 cells/cm^2^ on collagen (C4243, Merck), laminin (LN511-0202, Biolamina), fibronectin (1030FN, Bio Techne) or vitronectin (AF-140-09, Peprotech) coated T-flasks (0.25–1.00 μg/cm^2^) and cultured at 37°C, 5% CO_2_. Every 3–4 days, when a confluence of 60%–80% was reached, cells were harvested using 0.05% trypsin-EDTA (25300062, Gibco™, Thermo Fisher Scientific) or 10X TrypLE™ Select Enzyme (A1217701, Gibco™, Thermo Fisher Scientific) diluted (dilution 4:10) in PBS (10010023, Gibco™, Thermo Fisher Scientific). Cells were exposed to the enzyme for 5–15 min at 37°C, 5% CO_2_ and neutralized with serum or serum-free growth medium. Cells were then centrifuged (350 g, 5 min), the pellet was resuspended in serum or serum-free growth medium and a cell count was performed.

### 2.4 Microcarrier cultures

#### 2.4.1 Preparation of MCs

Cytodex-1^®^ (17044801, Cytiva), CellBIND^®^ (4,620, Corning), Synthemax II (3,535, Corning), SoloHill^®^ Plastic (P-221-020, Sartorius), SoloHill^®^ Plastic Plus (PP-221-020, Sartorius) and SoloHill^®^ Star Plus (SP-221-020, Sartorius) were prepared in accordance to manufacturer’s instructions. In brief, MCs were resuspended in PBS at 50 mL/g dry weight (DW) for at least 3 h prior to autoclave. After autoclaving (121°C, 20 min), the PBS was removed and the MCs were washed twice with serum or serum-free growth medium (30 mL/g DW). To perform washings, MCs were let to settle for 5–10 min and the supernatant was aspirated using a serological pipette. The MCs were then added to the serum or serum-free growth media for at least 1 h at 37°C, 5% CO_2_ prior inoculation.

#### 2.4.2 Coating of MCs with APCs

Laminin 511 (LN511-0202, Biolamina), fibronectin (1030FN, Bio Techne) or vitronectin (AF-140-09, Peprotech) were used to coat MCs. Laminin and fibronectin were directly used in their liquid form, whereas vitronectin was reconstituted in MilliQ water (1 mg/mL). To first evaluate the effect of APCs on cell attachment and growth (Figure 2), APCs were used as medium additives (vitronectin and laminin were used at 1 μg/mL and fibronectin at 10 μg/mL). For that, APCs were added to the serum-free growth medium containing the MCs and incubated for at least 1 h at 37°C, 5% CO_2_ prior inoculation. To investigate further the effect of vitronectin concentrations (Figures 4, 5), vitronectin was solely applied as a coating, proportionally to the surface area of MCs used (0, 16, 30 or 100 ng/cm^2^). For that, the amount of vitronectin needed was added to 50 mL of serum-free growth medium containing the MCs and incubated for 1 h at 37°C, 5% CO_2_. The medium was then removed and MCs were washed twice with serum-free growth medium (30 mL/g DW), as stated above. In addition, for Figure 4 and one condition of Figure 5, during medium exchanges an equivalent amount of vitronectin was used as a medium additive.

#### 2.4.3 Well plate cultures

For high-throughput media and MC screening (Figure 1A), a 24-well ultra-low attachment plate was used (174930, Nunclon™ Sphera™, Thermo Fisher Scientific). Cells were seeded at 1,800 cells/cm^2^ with a MC concentration of 10 cm^2^/mL. To allow MCs’ suspension, the plate was placed on an orbital shaker (88881102B, Thermo Fisher Scientific) at 70 rpm.

#### 2.4.4 Spinner flask cultures

Siliconised (SL2, Merck) glass spinner flasks with working volumes of 30 mL (356875, Wheaton®) or 100 mL (1965-00100, Bellco™) were used. Cells were seeded on MCs at 1,000–10,000 cells/cm^2^ with MC concentrations ranging from 5 to 80 cm^2^/mL. Stirring platforms (Cimarec™ Biosystem, Thermo Fisher Scientific) were set at 45 rpm for 100 mL spinner flasks or 70 rpm for 30 mL spinner flasks. Cell attachment was promoted by applying intermittent stirring for the first 2 days (5 min ON, 30 min OFF).

#### 2.4.5 Medium exchange

Every 2–3 days, a medium exchange was performed to replenish nutrients and remove waste metabolites. To perform a medium exchange, agitation was stopped for 5–10 min to allow MCs to settle, then 50%–75% supernatant was aspirated and replaced with fresh serum-free growth medium.

#### 2.4.6 Addition of MCs

For some conditions during the investigation on the effect of APCs (Figure 2), MCs were added mid-culture to achieve higher cell densities and to evaluate the bead-to-bead transfer. For these conditions, 1–3 MC additions were performed with a ratio of new:old MCs of 1:5. MCs were added when a confluence of 5,000–30,000 cells/cm^2^ was reached, usually in combination with a medium exchange. To promote bead-to-bead transfer, intermittent stirring (5 min ON, 30 min OFF) was applied for a day following the addition of new MCs.

#### 2.4.7 Harvesting cells from MCs

In order to assess cell purity as well as differentiation capacity post-processing, cells were harvested using a combination of enzymatic and mechanical techniques previously described by Nienow et al. (2014). Briefly, MCs were washed with PBS and incubated with 10X TrypLE™ Select Enzyme (A1217701, Gibco™, Thermo Fisher Scientific) diluted (dilution 4:10) in PBS. To facilitate cell detachment, MCs were exposed to stirring speeds of 90–120 rpm (100 mL spinner flask) or 120–900 rpm (30 mL spinner flask) for 15–30 min at 37°C, 5% CO_2_. The MC/cells suspension was then filtered using a 100 µm nylon mesh filter (SCNY00100, Merck) and centrifuged (350 g, 5 min). The pellet was then resuspended in serum or serum-free growth media and cell counts were performed.

### 2.5 Cell count (single cells and microcarriers)

For single cells obtained after thawing, 2D harvest or MC harvest, a sample was diluted (dilution 1:2) in a 0.4% trypan blue solution (T8154, Merck) and the cell count was determined with an automated counting chamber (Invitrogen™ Countess™ II FL, Thermo Fisher Scientific). For MCs cultures, an automated nucleocounter (NC200™, Chemometec) was used. The NC200™ requires the use of two reagents, a lysis buffer (910-0003, Chemometec) and a neutraliser (910-0002, Chemometec). After treatment, the sample is loaded on a cassette (941-0012, Chemometec) which is read by the NC200™ measuring the cell density.

### 2.6 Purity analysis

Cell purity was measured using a flow cytometer (MACSQuant10, Miltenyi Biotec) and using the method described by Messmer et al. (2023). Briefly, cells were stained using diluted (dilution 1:50) integrin α7 (ITGA7) (130-120-812, Miltenyi Biotec) and integrin α5 (130-122-076, Miltenyi Biotec) antibodies in 1% (w/v) BSA (A9418, Merck) in PBS solution. The percentage of SCs was calculated by gating for the ITGA5-/ITGA7+ population. These two markers were chosen as bSCs can be overgrown by fibro adipogenic progenitors (FAPs) which can be distinguished from bSCs as they are ITGA5+/ITGA7− (Messmer et al., 2023).

### 2.7 Differentiation

#### 2.7.1 2D differentiation

To assess 2D differentiation, cells were seeded in SFDM on 0.5% Matrigel (356238, Corning)-coated plates at a seeding of 100,000 cells/cm^2^ and cultured at 37°C, 5% CO_2_. Differentiation was observed for 3–5 days and till the differentiated cells started to detach from the plate.

#### 2.7.2 3D differentiation

To assess 3D differentiation, cells were seeded in RGD-functionalized alginate gel as previously described by Melzener et al. (2023). In brief, a 1.2–6 × 10^7^ cells/mL cell suspension in SFDM was prepared and mixed at a 1:1 ratio with the alginate solution (1.8 wt%). The gel was crosslinked using 100 mM CaCl_2_ for 5 min. After a wash, the gel was incubated in SFDM. A medium exchange was performed on day 3 and the formed bioartificial muscles (BAMs) were harvested on day 7 for further analysis.

### 2.8 Protein quantification

Proteins were extracted from the tissue samples using an alkaline high salt solution [600 mM NaCl; 100 mM NaOH (Mæhre et al., 2018)]. For protein quantification, the Micro BCA™ Protein Assay Kit (23235, Thermo Fisher Scientific) was used. Standards were prepared (2–200 μg/mL) by dilution of a 2 mg/mL BSA stock solution in water. Protein extracts were also diluted (1:50) in water. Duplicates of 100 µL diluted samples and standards were mixed with 100 µL of working solution (reagents MA, MB and MC mixed at a ratio of 50:48:2) in a 96-well plate and incubated for 2 h at 37°C. Absorbance at 570 nm was then measured using a plate reader (Victor X5 2030 Multilabel HTS Microplate Reader, Perkin Elmer).

### 2.9 ELISA for desmin, myosin and actin

In-house developed ELISA protocols were used to quantify desmin, slow myosin heavy chain and α-actin-1. For desmin, protein extracts were diluted to 0.5 μg/mL protein in carbonate coating buffer (pH 9.5), 5% RIPA lysis buffer (sc-24948, Santa Cruz Biotechnology) and 100 μg/mL bovine serum albumin (BSA; 126593, Sigma-Aldrich) and were added to an empty Nunc Maxisorp plate (M9410, Sigma-Aldrich). After 2 h of incubation, the plate was washed and blocked with 10 mg/mL BSA in Tris-buffered saline (TBS; 28358, Thermo Fisher Scientific). Samples were then stained with primary and secondary antibodies diluted in TBS + 0.1% Tween-20 and 2 mg/mL BSA incubated for 1 h each (Supplementary Table S1). For muscle specific slow myosin (MYH7) and α-Actin-1, capture antibodies were added to the plate in TBS and incubated overnight at 4°C. After washing the plate and blocking with 10 mg/mL BSA in TBS, samples were added at 40 μg/mL protein in TBS + 0.1% Tween-20 and 100 μg/mL BSA. After 2 h of incubation and washing, the detection antibodies (in-house biotinylated using Abcam Lightning Link biotinylation kit ab201795) and streptavidin-HRP were added and incubated for 1 h each (Supplementary Table S1). To visualize signals, TMB substrate (34028, Fisher Scientific) was added and incubated for 5–30 min (depending on the antigen) at room temperature. The reaction was stopped using 2 M sulfuric acid and the absorbances were measured at 450 nm using a plate reader (Victor X5 2030 Multilabel HTS Microplate Reader, Perkin Elmer). For all ELISAs dilution ranges of a reference muscle extract was used (from the rump of a 4 year old Belgian Blue bull) that contained average levels of slow and fast myosin, α-actin-1, desmin and actinin within a series of samples tested from very young to adult animals and from various anatomical locations.

### 2.10 Microscopy - brightfield and fluorescence imaging

EVOS™ Cell Imaging System (M5000, Invitrogen™) microscope was used to monitor 2D and MC cultures. For the latter, small samples of cultures (50–100 µL) were stained in a 48 or 96-well plate with 2 μg/mL of Hoechst (33342 392/440, Thermo Fisher Scientific) and 650 nM of Ethidium homodimer (EthD-1) (E1169, Invitrogen™). After 20 min of incubation at 37°C, 5% CO_2_, the cells/MC suspensions were imaged.

### 2.11 Cell growth

In order to characterize cell proliferation, cell count data were plotted using the logarithm LN (X/X_0_) over time, X = cell concentration on t and X_0_ = cell concentration on t = 0. The global doubling time was calculated using the maximum cell density reached, as follows:

$$Doubling\;time=\frac{\ln\left(2\right)*\left(t_{\max}-t_{0}\right)}{\ln\left(X_{\max}/X_{0}\right)}$$

### 2.12 Statistical analysis

All statistical analyses were performed using GraphPad Prism (v9.0.5). To evaluate significant differences between APCs on the global doubling time, the maximal confluence reached and the purity, one-way ANOVA combined with Tukey’s multiple comparisons test was used. Prior to analysis, outliers were identified and removed using the ROUT method with Q = 1%. For the growth data represented with the logarithmic curve, significant differences between the slopes were determined based on the analysis of covariance. Unless stated otherwise, error bars represent the standard deviation.

## 3 Results/discussion

### 3.1 Screening of serum-free media and MCs for bSCs growth

As cell attachment and growth can be influenced by parameters such as medium composition and MCs’ properties (charge, topography, stiffness, material), a high-throughput screening in a 24-well ultra-low attachment plate was carried out using an orbital shaker, to qualitatively assess bSCs attachment and growth for six different MCs and four media (Figure 1A). The MCs were used at 10 cm^2^/mL and the cell seeding was 1,800 cells/cm^2^.

In serum-free growth media, the best attachment and growth were observed with Cytodex 1, Synthemax II and Plastic Plus. These MCs are either positively charged (Cytodex 1, Plastic Plus) or contain a RGD peptide (Synthemax II). For the first group, the results obtained might be related to the difference of charges between the surface of the cell, which is known to be negatively charged (Weiss and Zeigel, 1971), and the positively charged MC resulting in an attractive force that may promote cell attachment. A study with embryonic stem cells showed better attachment on positively charged MCs compared to negatively charged ones (Chen et al., 2011). For Synthemax II, the attachment and growth could have been promoted by the RGD sequence present on the surface of the MC, which is one of the main domains responsible for cell adhesion (Derakhti et al., 2019; Ruoslahti and Pierschbacher, 1986). Using such an ECM peptide not only has the advantage of enhancing cell attachment, it also helps the maintenance of cell functionality and differentiation capacity (Derakhti et al., 2019). CellBIND showed mediocre performance, where cells were loosely attached and failed to proliferate. CellBIND MCs are negatively charged, resulting in a repulsive force which may impair cell spreading and consequently growth (Ahmad Khalili and Ahmad, 2015). Surprisingly, the Star Plus MC, also positively charged, failed to support cell attachment. This could be explained by its greater stiffness in comparison to Plastic Plus, also made of cross-linked polystyrene. Although electrostatic forces can potentially determine cell attachment, Hoshiba et al. (2018) showed that certain cells (HT-1080 and HeLa cells) adhered similarly on cationic, anionic, and nonionic substrates in serum-free medium, indicating that cell adhesion may not be mediated solely by surface charge. These results should therefore be interpreted with caution and other parameters should be considered, such as the adsorption of proteins on the MCs, which could influence cell attachment. Overall, the three serum-free media showed relatively comparable results, with the exception of the combination of mTeSR1 and Plastic Plus which failed to promote cell adhesion.

In serum, results were drastically different. The only MC successful in promoting cell attachment and growth was Cytodex 1. The main difference with the other MCs is the matrix composition. Cytodex 1 MCs are made of dextran and swell in contact with water (20 mL/g), whereas the others are polystyrene based MCs and do not swell (Supplementary Table S2). This could have an impact on the MC stiffness and hydrophilicity (Cytodex 1 MCs are probably softer and more hydrophilic), parameters that can influence cell attachment (Meng et al., 2017). However, no specific data are available on the stiffness and hydrophilicity of commercial MCs. Given the complexity of serum, it is possible that either attachment promoting or attachment inhibiting compounds interacted differently with the tested MCs. Previous studies already reported the growth of bSCs on Cytodex 1 (Andreassen et al., 2022; Verbruggen et al., 2018), however it was surprising that the rest of the MCs, especially CellBIND and Synthemax II, failed to promote cell adhesion and growth in serum containing medium. This discrepancy in results pertaining CellBIND and Synthemax II when compared to the Verbruggen study, may be attributed to the fact that they used a mix of fetal bovine and horse serum, whereas only fetal bovine serum was used here. A less specific sorting method was also used, which may have led to the presence of other cell types, such as FAPs, with the ability to attach and proliferate on CellBIND and Synthemax II. Subsequent studies were done in serum-free medium.

This first screening allowed the identification of a fewer number of promising conditions to be tried in spinner flasks, which are more representative of the environment in which cells will be grown at industrial scale. Additionally, as larger volumes are used, it is possible to perform cell counts and obtain quantitative data for more thorough comparisons. As a next step, the most promising combinations of media and MCs were tested (SFGM: Cytodex 1, Synthemax II and Plastic Plus; mTeSR1: Cytodex 1 and Syntemax II; individual data for each MC not shown). Results obtained in spinner flasks (Figure 1B) showed that bSCs grew significantly faster and reached higher densities in SFGM than in mTeSR1. Although similar growth rates were obtained between Cytodex 1, Synthemax II and Plastic Plus, a better cell distribution on the MCs and fewer cells in suspension were observed with Cytodex 1 (Figure 1C; Supplementary Figure S2). Cytodex 1 MCs are also easier to work with (transparent, which helps during microscopic observation) and are commercially available at large scale, making them more suitable for further experimentation.

Based on these results, the combination of SFGM and Cytodex 1 is the most promising combination for growing bSCs. Prior to investigating Cytodex 1 further, the cell detachment and differentiation phenotype were also checked (Figure 1D); these are important parameters to take into consideration when using non-degradable or non-edible MCs as the downstream process usually requires a MC free cell suspension. The proliferated bSCs were successfully detached and separated from the MCs while maintaining their ability to differentiate on 2D in SFDM (Figure 1D). Although cells showed ability to differentiate, part of them remained undifferentiated. This can be explained by the presence of distinct subpopulations, with some proliferative cells that fail to exit the cell cycle and some reserve cells that do not commit to myogenic differentiation (Melzener et al., 2024). Targeting ERK, NOTCH and RXR pathways has been shown to improve the proportion of cells participating in differentiation, leading to higher fusion index, level of myotube formation and muscle protein accumulation.

This first set of experiments showed that bSCs’ attachment and growth can be greatly affected by the MC and medium used. Although satisfying results were already obtained, the use of APCs could further enhance efficacy. Three ECM proteins were tested: vitronectin, laminin (5-1-1) and fibronectin. To better understand the effect of APCs, we pursued further experiments using Cytodex 1 in SFGM. In addition, laminin was used to determine if it could improve the results obtained with the Plastic MCs, which failed to provide cell attachment without APCs.

### 3.2 bSCs’ growth and purity enhancement with APCs

To understand the effect of APCs, cells from many different donors were cultured in spinner flasks in SFGM with Cytodex 1 MCs. The MCs were used either uncoated or were coated with vitronectin, laminin or fibronectin. The choice of materials was based on a previous review study conducted by our group (Bodiou et al., 2020). Animal-based and non-replicative materials, such as collagen and gelatin, were excluded from this study because of ethical and reproducibility concerns. Other non animal coatings, such as chitosan or poly-lysine, were evaluated on planar cultures but exhibited poor results and were therefore not considered suitable candidates for microcarrier cultures (data not shown). To evaluate the APC effects, the global doubling time, maximal confluence reached, cell morphology and purity were compared (Figure 2).

The use of vitronectin and laminin resulted in significantly higher growth rates of bSCs with lower global doubling times (Figure 2A). Average doubling times observed (48–55 h) are comparable to previous studies with bSCs in serum-free medium (Kolkmann et al., 2022; Stout et al., 2022). Doubling times of 12–72 h have been considered in techno-economic and life cycle analyses, but it was concluded that they did not have any significant impact on the cost, as the main contributors were culture medium and equipment (Ellersick et al., 2023; Gursel et al., 2022; Negulescu et al., 2023; Tuomisto et al., 2022). Nonetheless, it is still a parameter that can be improved and efforts are being made to optimize cells’ metabolism and growth rate (Messmer et al., 2023; Stout et al., 2023). Cell attachment and spreading is also enhanced when using APCs (Figure 2C). These results are expected as these proteins are typically found in ECM and are known to play important roles in SCs attachment and proliferation signaling pathways (Bentzinger et al., 2013; Ishii et al., 2018; Öcalan et al., 1988; Wilschut et al., 2010). Previous studies already reported improved growth of C2C12 cells and hESCs on MCs coated with proteins such as laminin, collagen and Matrigel (Bardouille et al., 2001; Chen et al., 2011). Recent research on animal-free APCs have also identified vegetal and fungal proteins with similar affinity to C2C12 cells and structures homologous to ECM proteins (Kong and Huang, 2023; Teo et al., 2023; Kong et al., 2023; Seo et al., 2023). These proteins helped improve cell attachment and proliferation, however media used in these studies still contained fetal bovine serum. It would therefore be interesting to test them in a serum-free medium to develop a more ethical and reproducible bioprocess. Although fibronectin coating resulted in a higher maximal confluence (Figure 2B), in the highly confluent samples (>100,000 cells/cm^2^) this led to formation of MC aggregates and detachment of cells (Supplementary Figure S3). These situations can increase the complexity of unit operations, such as medium exchange or harvesting, and it remains to be seen if detachment affects the functionality of these cells. Lastly, purity results showed that using vitronectin and laminin significantly contributed to maintaining a higher percentage of bSCs in comparison to fibronectin (Figure 2D) suggesting that the latter favors the growth of other cell populations than bSCs. These results are in-line with a previous study using bSCs on 2D coated surfaces, in which a higher purity was achieved when using laminin compared to fibronectin (Messmer et al., 2023). The decrease in bSCs’ purity on fibronectin, is attributed to an overgrowth of FAPs. Thus, despite the higher confluence reached with fibronectin, in order to enhance cell attachment and growth and to maintain a high SCs purity, it is preferable to use vitronectin or laminin as APCs.

It is worth noting here there is some donor to donor variability at play too. Beside finding a suitable APC, the variability between donors was also evaluated (Figure 3). For each APC, some donors were growing faster than others (Figures 3A–D). Analysis of the variance on the donor and APC showed that both contributed significantly to the variance of the global doubling time. Nonetheless, the coefficient of variation observed with APCs was lower compared to uncoated MCs (Figure 3E). In addition, using an APC, and more specifically vitronectin, helped to maintain higher bSCs purity throughout the culture, regardless of the donor used.

Next, we wanted to investigate if the use of APC could improve the attachment and growth on MCs that previously failed to provide attachment and growth when used uncoated. For this, Plastic MCs that previously showed neither attachment nor growth in the 2D screening (Figure 1A), were coated with laminin. The cells grew similarly on coated Plastic when compared to Cytodex 1, but failed to continue growing after fresh MCs were added, despite the seemingly successful bead-to-bead transfer as judged by the 95% colonization of beads at the end of culture (Supplementary Figure S4). Cells grown on laminin-coated Plastic MCs maintained their ability to differentiate.

### 3.3 Investigation of vitronectin concentration and its mode of application

Although vitronectin and laminin showed similar cell attachment and growth promoting effects, vitronectin resulted in less variability with regards to bSCs population purity and is a smaller molecule to manufacture (Beck et al., 1990; Suzuki et al., 1984). We therefore continued our research with vitronectin. In the experiments performed so far, APCs were used at relatively high concentrations during coating and were also added during medium exchanges to maximize their beneficial effect. However, applying this method resulted in varying APCs concentration per surface area of MC, depending on the MCs concentration (vitronectin: 1 μg/mL ⇔ 20–200 ng/cm^2^; laminin: 1 μg/mL ⇔ 20–200 ng/cm^2^; fibronectin: 10 μg/mL ⇔ 200–2,000 ng/cm^2^). To better understand the role of vitronectin, we investigated the effect of its concentration (Figure 4) and then determined if the mode of vitronectin addition, i.e., as a medium component *versus* MC coating, had an effect on cell attachment and growth (Figure 5). For the former, bSCs were cultured on MCs (40 cm^2^/mL) coated with varying concentrations of vitronectin, using 100 mL spinner flasks and SFGM.

Four vitronectin concentrations were tested (0, 16, 30 and 100 ng/cm^2^). During each medium exchange (day 3 and 5), the same concentration of vitronectin was re-introduced. Cells were able to attach and grow in all concentrations of vitronectin. A significant difference in growth rate was observed between 0 and 100 ng/cm^2^ (Figure 4A). There was no significant difference with regard to cell purity at the end of the culture (Figure 4B). Post processing, cells differentiated successfully on 2D for all vitronectin concentrations (Figure 4C). No noticeable differences were observed in 3D differentiation (Figure 4D). The differentiated samples consisted of 20%–30% protein, which is in accordance with the *in vivo* protein content of bovine muscle tissue (Choi et al., 2023; Honig et al., 2022). When compared to the beef meat lysate, the differentiated samples showed a higher percentage of desmin and lower levels of myosin and actin. This could be attributed to an early state of differentiation. Cells were indeed only differentiated for 7 days, which is a relatively short period in comparison to some other studies that cultured 3D constructs for over 20 days (Aguanno et al., 2019; Rhim et al., 2007). These results suggest that regardless of the vitronectin concentration used, cells can attach, proliferate and differentiate similarly. Therefore, for the next experiments investigating the mode of application, the lowest vitronectin concentration of 16 ng/cm^2^ was used. For this, cells from three donors were cultured in 100 mL spinner flasks and with a MC concentration of 60 cm^2^/mL.

Finally, to further investigate the effect of vitronectin, its addition during medium exchanges was omitted and it was only used as MC coating. No significant differences in terms of growth (Figure 5A), purity (Figure 5B), 2D (Figure 5C) or 3D differentiation (Figure 5D) were observed. As in the meat lysate, muscle-specific proteins actinin and alpha-actin-1 were found, however myosin and myoglobin were absent. Here too, differentiation was only run for 7 days, which might have not been enough to provide all muscle-specific proteins.

In summary, we showed that vitronectin can be used at 16 ng/cm^2^ on Cytodex 1 and that its addition during medium exchanges can be omitted without affecting cell growth, purity or differentiation. In addition to offering a better understanding and control over the parameters affecting cell attachment and growth, its use only as a coating rather than medium additive during medium exchanges, also contributes to a significant cost reduction, namely, 3-50 fold decrease, depending on the MC concentration.

These results, although applicable in a dynamic environment (spinner flasks), are yet to be confirmed at larger scales and in bioreactors. Cell attachment, growth and detachment are three of the main challenges associated with MC based cultures at larger scales. To promote cell attachment and growth while maintaining cell purity in bioreactors, the use of an APC, and more specifically vitronectin, may likely be one of the simplest ways to do so, rendering our findings directly translatable. However, since spinner flasks are not ideal scale-down models of bioreactors and an increase in hydrodynamic forces is expected in larger scales (Berry et al., 2016; Jossen et al., 2014; Julaey et al., 2016), the APC concentration might need to be adjusted.

## 4 Conclusion

bSCs can efficiently grow on MCs in a serum-free medium with the help of vitronectin as a coating. Other APCs tested were not as effective. Fibronectin reduced the growth and the purity of the bSCs population, and laminin resulted in a higher variability in the bSCs purity. The mode of application and concentration of vitronectin are also important as they may complicate the processing and increase the associated costs. Vitronectin can be used solely as a coating and its addition during medium exchanges can be omitted, without any impact on cell growth, purity or subsequent myogenic differentiation. All of the above contribute to an effective way of sustainably scaling up bSCs production under serum-free conditions for the purpose of meat cultivation.

## Data Availability

The original contributions presented in the study are included in the article/Supplementary Material, further inquiries can be directed to the corresponding author.
