# Supplementary material for: Attachment promoting compounds significantly enhance cell proliferation and purity of bovine satellite cells grown on microcarriers in the absence of serum
Source: Front Bioeng Biotechnol. 2024 Nov 1;12:1443914. doi: 10.3389/fbioe.2024.1443914 (PMC11563957; doi:10.3389/fbioe.2024.1443914)
Supplement: Supplementary file 1 [file Table1.DOCX]

Supplementary Table 1: Antibodies used for ELISA.

| **Target** | **Tag** | **Concentration** | **Species** | **Product reference** |
| --- | --- | --- | --- | --- |
| Desmin | - | 1:3000 | Rabbit monoclonal | ab227651, Abcam |
| Anti-Rabbit | HRP | 0.25 µg/ml | Goat | ab6721, Abcam |
| Slow Myosin | - | 1.5 µg/ml | Mouse monoclonal (NOQ7.5.4D) | MAB1628, Merck |
| Slow Myosin | biotin | 1 µg/ml | Rabbit polyclonal | ab197687, Abcam |
| a-Actin-1 | - | 1:1200 | Mouse monoclonal (5C5) | A2172, Sigma-Aldrich |
| a-Actin-1 | biotin | 1.68 µg/ml | Rabbit polyclonal | ab97378, Abcam |
| Streptavidin | HRP | 1 mg/ml | - | ab7403, Abcam |
