# Supplementary material for: Attachment promoting compounds significantly enhance cell proliferation and purity of bovine satellite cells grown on microcarriers in the absence of serum
Source: Front Bioeng Biotechnol. 2024 Nov 1;12:1443914. doi: 10.3389/fbioe.2024.1443914 (PMC11563957; doi:10.3389/fbioe.2024.1443914)
Supplement: Supplementary file 2 [file Image4.PDF]

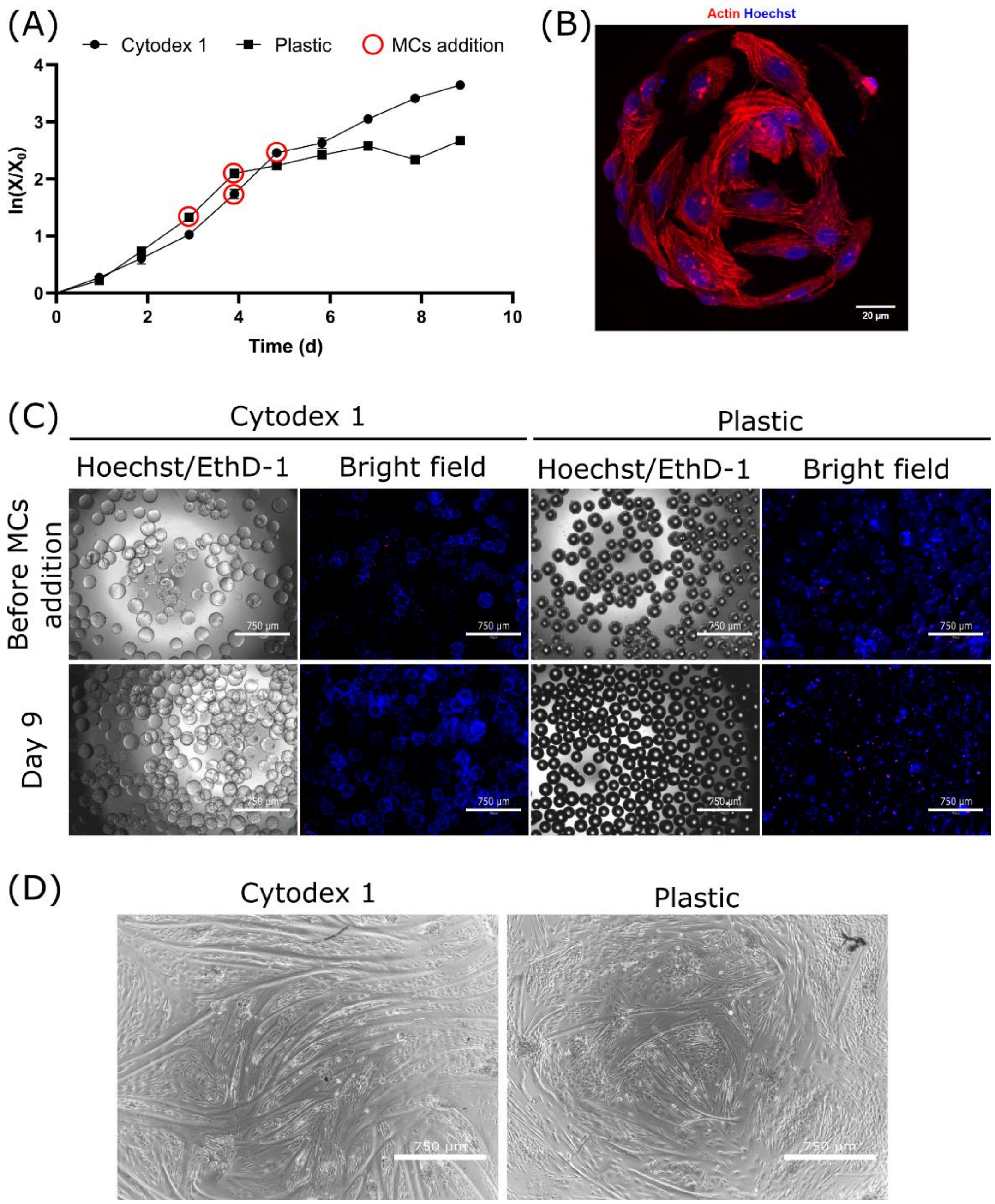

<u>Supplementary Figure 4:</u> **(A)** Logarithmic growth of bSCs cultured on Cytodex 1 and Plastic MCs coated with Laminin in SFGM (MCs added on day 4 and 5 for Cytodex 1, and on day 3 and 4 for Plastic ; for both the MC concentration was increased from 10 to 20 cm^2/ml and then from 20 to 40 cm^2/ml. **(B)** Actin and Hoechst staining of Plastic MCs coated with laminin on day 7 of culture. **(C)** Fluorescent images (Hoescht/EthD-1) and bright field images (magnification x4; scale bar=750 µm) of bSCs cultured on Cytodex 1 and Plastic MCs coated with Laminin in SFGM (MCs added on day 4 and 5 for Cytodex 1, and on day 3 and 4 for Plastic; for both the MC concentration was increased from 10 to 20 cm^2/ml and then from 20 to 40 cm^2/ml). **(D)** Bright field images (magnification x4; scale bar=750 µm) of 2D differentiation of bSCs after proliferation on laminin coated Cytodex 1 and Plastic MCs.
