# Supplementary material for: Attachment promoting compounds significantly enhance cell proliferation and purity of bovine satellite cells grown on microcarriers in the absence of serum
Source: Front Bioeng Biotechnol. 2024 Nov 1;12:1443914. doi: 10.3389/fbioe.2024.1443914 (PMC11563957; doi:10.3389/fbioe.2024.1443914)
Supplement: Supplementary file 3 [file Image2.PDF]

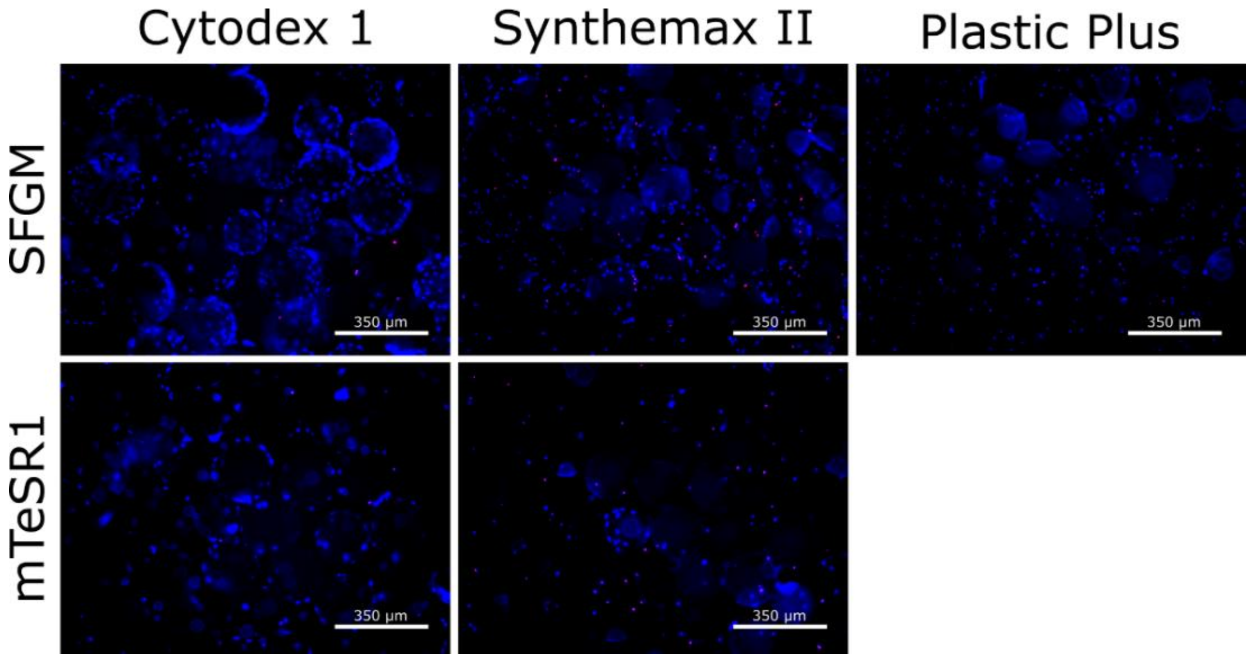

<u>Supplementary Figure 2:</u> Day 6 fluorescent images (Hoescht/EthD-1; magnification x10; scale bar=350 µm) of bSCs grown in two serum-free growth media (SFGM and mTeSR1) and on three different MCs (Cytodex 1, Synthemax II and Plastic Plus). The experiment was performed in 30 ml spinner flasks at 10 cm^2/ml and cell seeding density of 5,000 cells/cm^2.
