# Supplementary material for: Attachment promoting compounds significantly enhance cell proliferation and purity of bovine satellite cells grown on microcarriers in the absence of serum
Source: Front Bioeng Biotechnol. 2024 Nov 1;12:1443914. doi: 10.3389/fbioe.2024.1443914 (PMC11563957; doi:10.3389/fbioe.2024.1443914)
Supplement: Supplementary file 4 [file Image3.PDF]

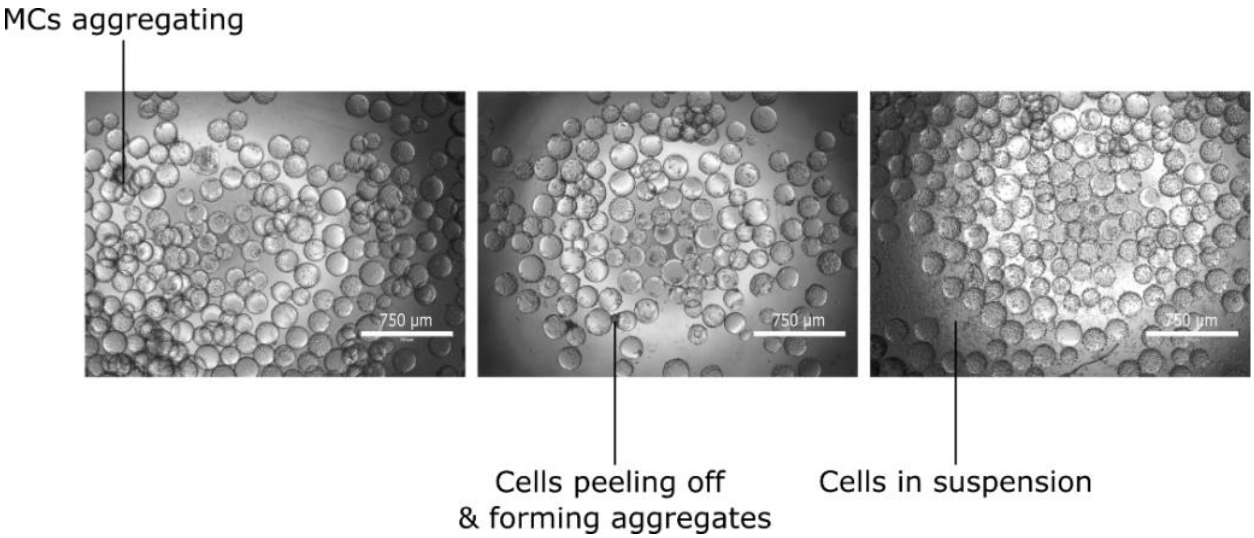

<u>Supplementary Figure 3:</u> Bright field images (magnification x4; scale bar=750 µm) of bSCs cultured on Cytodex 1 coated with fibronectin in SFGM.
