# Supplementary material for: Attachment promoting compounds significantly enhance cell proliferation and purity of bovine satellite cells grown on microcarriers in the absence of serum
Source: Front Bioeng Biotechnol. 2024 Nov 1;12:1443914. doi: 10.3389/fbioe.2024.1443914 (PMC11563957; doi:10.3389/fbioe.2024.1443914)
Supplement: Supplementary file 5 [file Table2.DOCX]

Supplementary Table 2: Properties of commercial MCs

| **Microcarriers** | **Manufacturer** | **Matrix** | **Surface coating** | **Surface charge** | **Diameter (µm)** | **Specific density** | **Porosity** | **Specific surface area (cm^2^/g DW)** |
| --- | --- | --- | --- | --- | --- | --- | --- | --- |
| Cytodex 1 | GE Healthcare | Cross-linked dextran | DEAE | + | 147-248 | 1.03 | Non-porous | 4400 |
| CellBIND | Corning | Polystyrene | CellBIND | - | 125-212 | 1.02 | Non-porous | 360 |
| Synthemax II | Corning | Polystyrene | Synthemax II | None | 125-212 | 1.02 | Non-porous | 360 |
| Plastic | SoloHill, Sartorius | Polystyrene | None | + | 90-150 | 1.02-1.03 | Non-porous | 360 |
| Plastic Plus | SoloHill, Sartorius | Polystyrene | None | + | 125-212 | 1.034-1.046 | Non-porous | 360 |
| Star Plus | SoloHill, Sartorius | Polystyrene | None | + | 125-212 | 1.02-1.03 | Non-porous | 360 |
