# Supplementary material for: Attachment promoting compounds significantly enhance cell proliferation and purity of bovine satellite cells grown on microcarriers in the absence of serum
Source: Front Bioeng Biotechnol. 2024 Nov 1;12:1443914. doi: 10.3389/fbioe.2024.1443914 (PMC11563957; doi:10.3389/fbioe.2024.1443914)
Supplement: Supplementary file 6 [file Image1.PDF]

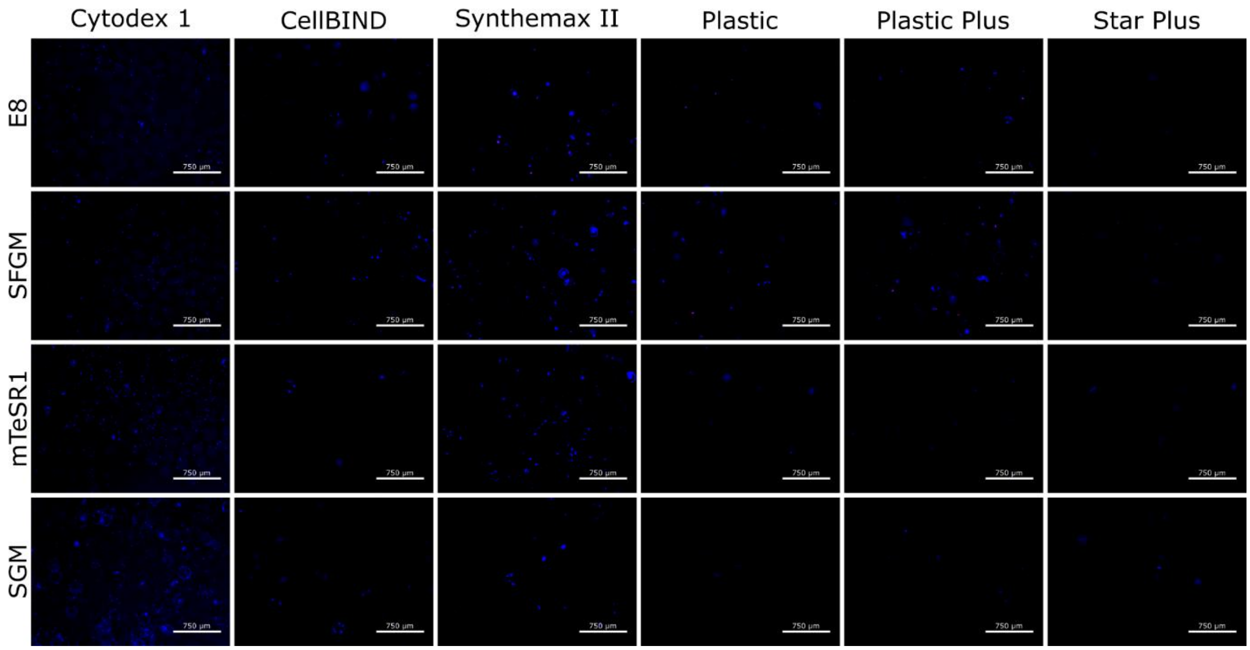

<u>Supplementary Figure 1:</u> Day 6 fluorescent images (Hoescht/EthD-1; magnification x4; scale bar = 750 μm) of bSCs grown on 6 different microcarriers (Cytodex 1, CellBIND, Synthemax II, Plastic and Plastic Plus) and four media: three serum-free media (E8, SFGM and mTeSR1) and one serum based (SGM). The experiment was performed in a 24-well ultra-low attachment plate on an orbital shaker at 10 cm^2/ml and cell seeding density of 1,800 cells/cm^2.
